# The acceptability mass administrations of anti-malarial drug as part of targeted malaria elimination in villages along the Thai–Myanmar border

**DOI:** 10.1186/s12936-016-1528-7

**Published:** 2016-09-27

**Authors:** Ladda Kajeechiwa, May Myo Thwin, Paw Wah Shee, Nan Lin Yee, Elvina Elvina, Peapah Peapah, Kyawt Kyawt, Poe Thit Oo, William PoWah, Jacqueline Roger Min, Jacher Wiladphaingern, Lorenz von Seidlein, Suphak Nosten, Francois Nosten

**Affiliations:** 1 Shoklo Malaria Research Unit, Mae Sot, Thailand; 2 Mahidol Oxford Research Unit, Faculty of Tropical Medicine, Mahidol University, Bangkok, Thailand; 3 Nuffield Department of Medicine, Centre for Tropical Medicine and Global Health, University of Oxford, Oxford, UK

**Keywords:** Malaria, Mass drug administration, Targeted malaria control, Community engagement, Social mobilisation, Acceptance, Knowledge, Behaviour

## Abstract

**Background:**

A targeted malaria elimination project, including mass drug administrations (MDA) of dihydroartemisinin/piperaquine plus a single low dose primaquine is underway in villages along the Thailand Myanmar border. The intervention has multiple components but the success of the project will depend on the participation of the entire communities. Quantitative surveys were conducted to study reasons for participation or non-participation in the campaign with the aim to identify factors associated with the acceptance and participation in the mass drug administrations.

**Methods:**

The household heads in four study villages in which MDAs had taken place previously were interviewed between January 2014 and July 2015.

**Results:**

174/378 respondents (46 %) completed three rounds of three drug doses each, 313/378 (83 %) took at least three consecutive doses and 56/378 (15 %) did not participate at all in the MDA. The respondents from the two villages (KNH and TPN) were much more likely to participate in the MDA than respondents from the other two villages (HKT and TOT). The more compliant villages KNH and TPN had both an appearance of cohesive communities with similar demographic and ethnic backgrounds. By contrast the villages with low participation were unique. One village was fragmented following years of armed conflict and many respondents gave little inclination to cooperate with outsiders. The other village with low MDA coverage was characterised by a high percentage of short-term residents with little interest in community interventions. A universal reason for non-participation in the MDA applicable to all villages was an inadequate understanding of the intervention.

**Conclusions:**

It is unlikely that community engagement can unite fragmented communities in participating in an intervention, which benefits the community. Understanding the purpose and the reasons underlying the intervention is an important pre-condition for participation. In the absence of direct benefits and a complete understanding of the indirect benefits trust in the investigators is critical for participation.

## Background

Economic development, deforestation and on-going malaria control activities including the distribution of insecticide-treated bed nets (ITNs) and improved management have reduced malaria prevalence to historically low levels in the Greater Mekong Subregion (GMS). The recent emergence and spread of artemisinin resistance in Western Cambodia is a threat for the effective treatment of falciparum malaria [1]. In the absence of alternative first-line anti-malarial drugs increasing falciparum malaria related morbidity and mortality can be expected. The interruption of malaria transmission is the remaining best strategy to stop the spread of artemisinin-resistant *Plasmodium falciparum,* which requires the elimination of all falciparum infections including the submicroscopic parasite reservoir. The large majority of parasitaemia people in low transmission settings have no clinical signs or symptoms of malaria hence screening with rapid diagnostic tests, microscopy and even standard PCR misses a proportion of infections [2]. Targeted malaria elimination includes mass drug administrations (MDA) for the presumptive treatment of all residents in foci of high sub-microscopic infections in addition to improved case management by village health workers and the distribution of ITNs.

Mass administrations of anti-malarial drugs have been recorded and reported for more than a century [3]. Well-conducted campaigns have interrupted malaria transmission for extended periods [4] and permanently in an island setting (Aneityum, Vanuatu) [5]. Public health interventions depend on the goodwill and support of the community [6]. Mass administrations of anti-malarial drugs differ fundamentally from other interventions in that the participation of the entire community is essential for success. Non-participants can become a residual reservoir for infections, continue to transmit malaria and thus prevent elimination. There has been extensive research on the pharmacokinetics, dynamics, and efficacy of various anti-malarial drug regimens [7]. Less is known about the factors, which influence community acceptance or refusal to participate in mass administrations of anti-malarial drugs.

Newby and co-workers conducted a systematic literature review of mass administrations of anti-malarial drugs in 2014 [3]. Very few of the articles provided detailed descriptions of knowledge, attitudes and perceptions towards mass administrations or a description of efforts to engage the community and increase participation. Examples for continued research on the acceptance of anti-malarial mass drug administration campaigns come from Aneityum Island [8, 9] and The Gambia [10–12]. One such report described a quantitative survey following a mass administration of anti-malarials conducted in The Gambia in 1998/99 [10]. Individuals who believed in the importance of the MDA and those who were aware that a high level of participation was needed for the MDA to be successful were more likely to participate.

Understanding that the purpose of the MDA was to reduce malaria, knowledge of the fact that malaria is transmitted by mosquitoes and awareness of the clinical signs of malaria were associated with participation. Individuals who discussed the MDA with other villagers and those who attended the sensitization meeting were also more likely to participate than those who did not. In The Gambia women were significantly more likely than men to participate in the mass drug administrations. The investigators concluded that better information could lead to increased participation in their target populations in rural West Africa. In 2013, 14 years after the campaign, social scientists re-visited the study site in The Gambia and interviewed the population about this and subsequent mass drug administrations targeting trachoma transmission [11]. This later qualitative study found that timing of the campaign, accurate information on the procedures, drug regimen, and possible side effects were critical for participation. The authors concluded that continuous sensitization meetings may be needed to achieve high coverage. In 2014 a second mass administration of anti-malarial drugs was conducted in The Gambia. A combined quantitative and qualitative study explored the motivations and circumstances for non-uptake and non-adherence [12]. This time the most frequently mentioned reasons for non-participation were mobility/travel, fear of drug reactions, inconvenience and insufficient information.

A targeted malaria elimination project is currently underway in villages along the Thailand–Myanmar border. Quantitative surveys were conducted to study reasons for participation or non-participation in the drug administration campaign with the aim to identify factors associated with acceptance of the complete drug administration, three rounds of three doses of anti-malarials, part of the drug administration or no participation at all.

## Methods

### The villages

Four Karen villages (HKT, KNH, TOT, and TPN) located within 10 km of the Thailand Myanmar border were selected for inclusion in a targeted malaria elimination project based on the prevalence of *P. falciparum* parasitaemia detected by high volume ultrasensitive real time polymerase chain reaction (uPCR) [2, 13]. Briefly the overall population in the four villages is 2377 (HKT 908, KNH 349, TOT 745, and TPN 375). Using uPCR 520 (34 %) of 1536 people who participated in an initial survey were infected [*P. falciparum* 87/1536 (6 %), *Plasmodium vivax* 230/1536 (15 %), mixed infections 21/1536 (1 %), and *Plasmodium species* 182 (12 %)]. 142/152 (93 %) participants with *P. falciparum* infections and 309/323 (96 %) with *P. vivax* infections were asymptomatic on the day of the survey [13]. The study population consists of three major ethnicities (Burman, Pow Karen, and Sgaw Karen).

Like many ethnic groups in Myanmar, the Karen, have been involved in conflicts with the central government since independence in 1949. The Karen National Liberation Army (KNLA) was the main force in Karen State until 1994 when other Karen groups were formed such as the Democratic Karen Buddhist Army (DKBA). Karen villagers in the conflict zones of Eastern Karen State have usually no representation in the central Myanmar government nor does the central government have a stable presence in the study villages. This complex and unstable geo-political situation has affected the lives of villagers in this area and is strongly felt in villages where sometimes opposing groups continue to coexist. Despite recent cessation of armed hostilities, these factors have contributed to the deterioration of the social fabric. This was most evident in TOT where strong contingents of KNLA and DKBA rule different sections of the village. HKT used to be a small settlement but has grown into trading post over the last decade with a relatively large population of shopkeepers and their relatives, none of whom are Karen.

### The intervention

**Fig. 1:**
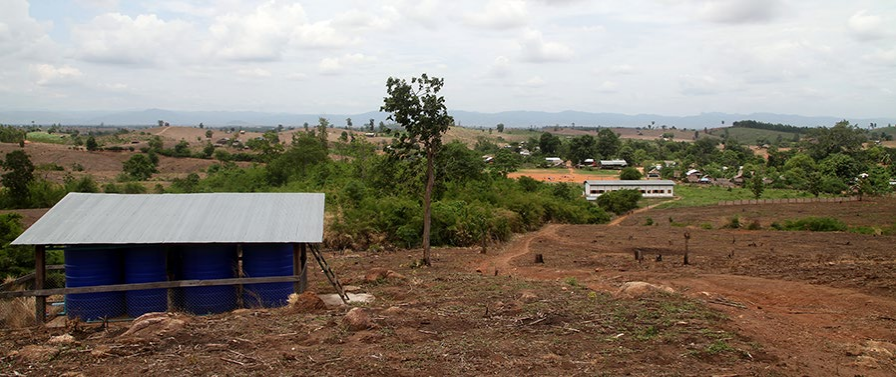
Three water storage tanks were installed by the study team covered by a corrugate iron roof, which are a part of the improved water supply system provided as a community incentive to the village in the background

After approval from regional and village leaders a series of village meetings were conducted. All members of the target population were invited to participate in the drug administration with the exception of infants under 6 months of age and pregnant women in the 1st trimester. To assure that pregnant women in the 1st trimester did not participate the entire population was reminded of the exclusion criteria at each meeting. Women unsure about their pregnancy status were offered rapid pregnancy tests free of cost. The methods and purpose of the campaign were explained and questions were answered. To engage the community in the study which included multiple blood draws [13] besides the drug administration the investigators pledged to provide a community incentive. Based on discussions with the villagers the study team facilitated the installation of an improved water supply system for the benefit of the entire village (Fig. 1). The primary health care centres where the intervention was delivered were rehabilitated and refurbished or newly established if no appropriate structure was available. House to house visits were conducted to inform and invite all eligible residents to participate in the drug administration. A multifaceted programme was offered to the villagers at a central place, usually the primary health care centre explaining the transmission and pathogenesis of malaria as well as the rational and methods of the planned drug administration as well as other methods to prevent malaria. These small group meetings included a drama-show scripted by the community engagement team and a display of posters designed by the team. Additional projects such as meetings and children activities were organised in the schools. Participants received snacks and refreshments during the meetings. No individual monetary incentives were provided for participants in the MDA.

**Fig. 2:**
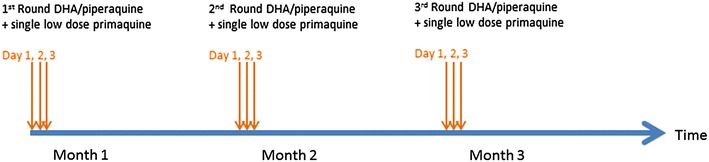
A schematic representation of treatment regimen in targeted malaria elimination (TME)

**Fig. 3:**
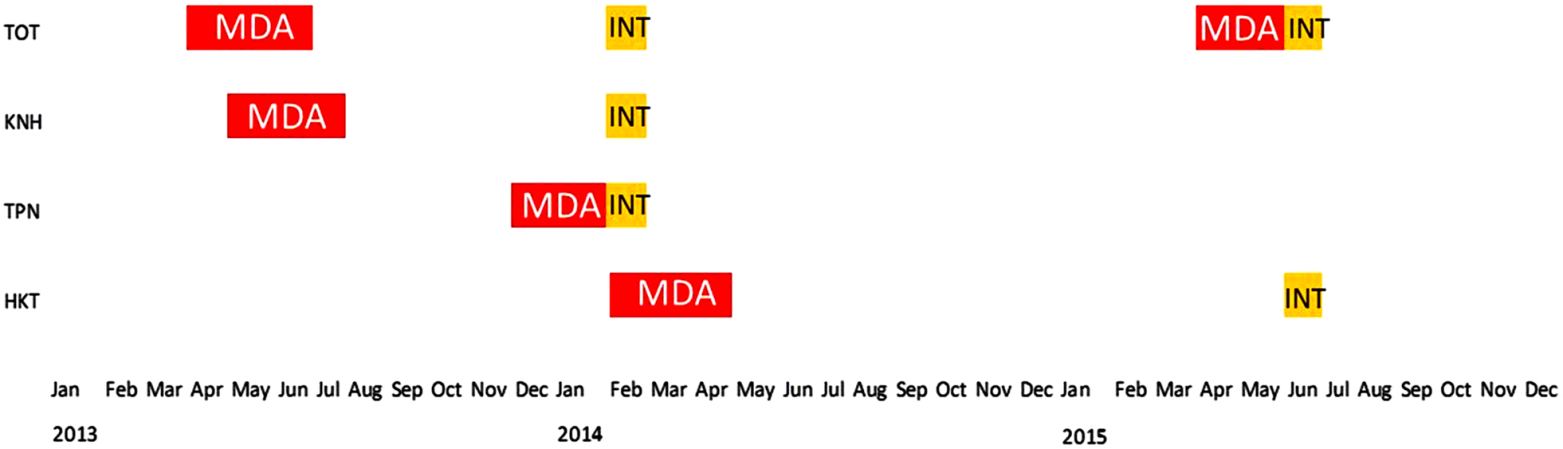
The approximate timing of the interviews (INT) in relation to mass drug administrations (MDA). The interviews were usually completed in the less than a week while the completion of three rounds of MDAs required 3 months

Three rounds of the anti-malarial drugs were given 1 month apart. Each round consisted of three daily doses of dihydroartemisinin/piperaquine plus a single low dose primaquine (0.25 mg/kg). The first MDAs in TOT and KNH took place between April and July 2013 and the second set between December 2013 and April 2014. The drugs were administered under direct observation. The drug regimen is illustrated in Fig. 2. Following the drug administrations the residents in intervention and control villages were under fever surveillance for a 24 months period. All villagers were asked to participate in 3-monthly surveys to detect submicroscopic parasitaemia by uPCR. Between surveys a village health worker diagnosed using rapid diagnostic tests and treated malaria episodes. Due to lower than expected coverage the residents of TOT were offered an opportunity to participate in a second MDA between April and June 2015. The timing of the interviews in relation to the MDAs is illustrated in Fig. 3.

### The interviews

A structured instrument was developed, based on the questionnaire used in The Gambia in 1999 [10]. The investigators translated the questionnaire into local languages and adapted the instrument to local circumstances. After piloting and finalization of the questionnaire interviewers who were not members of the sensitisation team and did not participate in other aspects of the intervention, conducted the interviews. The aim was to interview the household heads or their representatives in each household in each study village. In case the household head could not be interviewed after repeated attempts the next senior household member was interviewed. The first sets of interviews were conducted in TOT, KNH, and TPN in January and February 2014. A second set of interviews was conducted in June 2015 in HKT and in TOT following the completion of a second MDA in that village. The timing of the interviews in relation to the drug administration campaigns is illustrated in Fig. 3.

### Data management and analysis

The responses were recorded on paper questionnaires, translated into English and single entered in Microsoft Access (Access version 14, Microsoft, Redmond, Washington, USA). The number of DHA/piperaquine doses received by each respondent was based on the data collected by the drug administration team. The files were linked using unique identification numbers assigned to each household member and used by both interviewer and drug administration teams.

For the purposes of this acceptance study respondents who did not participate in the MDA (i.e. did not take a single dose) were defined as non-participants. This group of non-participants may have been absent or completely refused participation. In a secondary analysis respondents who did not participate in a single complete round (three consecutive doses) necessary to clear parasitaemia completely were considered non-participants. People who took at least one dose but less than nine doses were defined as incomplete participants, and people who took three rounds of three doses each (i.e. nine doses) were defined as complete participants. The ingestion of a single low dose primaquine with each round of DHA-piperaquine was not included in the definitions of participation.

Normally distributed data were analysed using the Chi squared test or Fisher’s exact test as appropriate. Non-parametric continuous data were analysed using Kruskal–Wallis equality-of-populations rank test or Mann–Whitney test as appropriate. Considering the number of variables and hypotheses tested only a conservative p < 0.01 was considered significant. A logistic regression model was constructed to identify variables independently associated with the participation in three rounds of three doses DHA piperaquine (i.e. complete participation). For this purpose the respondents were re-categorised into complete participants who took nine doses DHA-piperaquine or respondents who took less than nine doses. Only responses collected during the first set of interviews in TOT in 2014 were included in the models due to the high correlation between sequential responses in the same household. The final model was adjusted for the significant variables in the univariate analysis. All analyses were performed using Stata, version 14 (StataCorp, College Station, TX, USA).

## Results

### Background and demographics

388 respondents were interviewed. The participation status in the mass drug administration of 378/400 respondents (97 %) was documented in the database. Overall 313/378 respondents (83 %) took at least three consecutive doses of the anti-malarials required for the complete clearance of parasites. 174/378 respondents (46 %) completed three rounds of three drug doses each, 56/378 (15 %) did not participate at all, and the remaining 148 (39 %) respondents participated but did not complete the full course of 9 doses (Table 1).

**Table 1:** Demographics, education and profession of respondents in relation to participation

	No MDA %		Incomplete MDA %		Complete MDA %		Total		p value*
Number respondents	56	15 %	148	39 %	174	46 %	378	100 %	NA
Sex									
Female	39	70 %	111	75 %	125	72 %	284	73 %	
Male	17	30 %	36	25 %	46	26 %	107	26 %	
No answer	0		1	1 %	3	2 %	4	1 %	0.99
Age (mean in years)	39		39		40		39		0.79**
Village									
HKT	34	61 %	52	35 %	37	22 %	123	33 %	
TOT^a^	19	34 %	59	40 %	36	21 %	114	30 %	
KNH	2	4 %	15	10 %	58	33 %	75	20 %	
TPN	1	2 %	22	15 %	43	25 %	66	18 %	<0.001
Ethnicity									
Sgaw	23	41 %	76	51 %	73	42 %	172	46 %	
Paw	11	20 %	31	21 %	66	38 %	108	29 %	
Burman	21	38 %	38	26 %	29	17 %	88	23 %	
Other	1	2 %	2	1 %	5	3 %	8	2 %	
No answer	0		1	1 %	1	1 %	1	%	0.002
Literacy									
Yes	36	64 %	82	55 %	105	60 %	223	59 %	0.457
Profession									
Farmer	33	59 %	94	64 %	110	63 %	237	63 %	0.818
Other	7	13 %	35	24 %	39	22 %	81	21 %	NA
Shopkeeper	15	27 %	14	9 %	13	7 %	42	11 %	0.008
Forest worker	1	2 %	5	3 %	12	7 %	18	5 %	0.349

Responses are sorted by totals; with the exception of the first row percentages % refer to columns not rows

*MDA* mass drug administration, *NA* not applicable

* Chi squared test or Fisher’s exact test

** Kruskal–Wallis equality-of-populations rank test

^a^2014

**Fig. 4:**
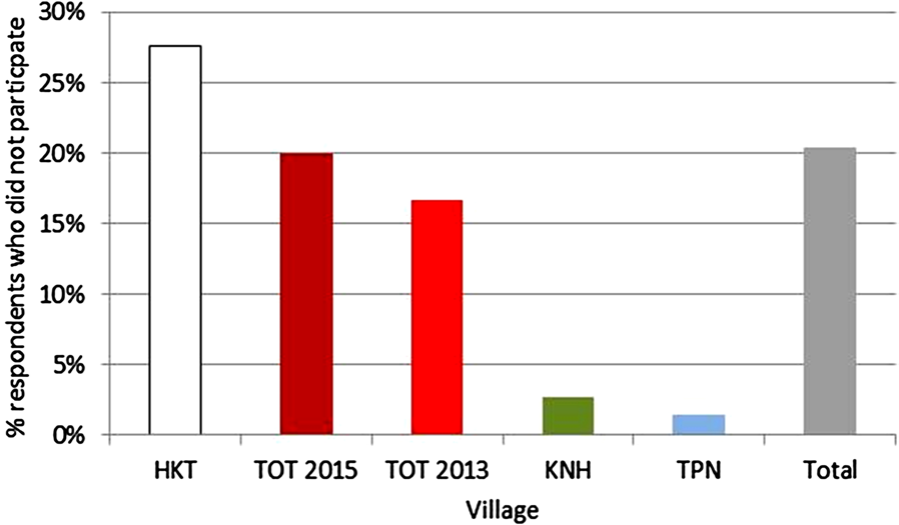
Percentage of respondents who did not participate in the MDA by village (and year of mass drug administration in TOT)

The participation of respondents in the MDA varied significantly between villages (Fig. 4). In KNH 72/75 (96 %) of the respondents took at least three consecutive doses of anti-malarials, in TPN 63/66 (95 %), and in HKT 87/123 (81 %). During the first drug administration in 2013 in TOT 91/114 (80 %) respondents took at least three doses. During a second MDA in 2015 the number of respondents who took at least three doses was not significantly lower 76/105 (73 %; p = 0.2).

The respondents identified with four main ethnicities Sgaw, Burman, and Poe. A significantly larger proportion of Burmans (21/88, 24 %) did not participate in the MDA than members of other ethnicities (Sgaw 23/172, 14 %; Paw 11/108; 10 %; p < 0.002). The ethnicity varied between villages; the large majority of the respondents in TOT (88 %; 100/114) were Sgaw and Burman in HKT (58 %; 71/123). Literacy was overall 59 % (223/378) but varied significantly between villages. 75 % (56/75) of the respondents in KNH, 73 % (95/131) in HKT, 66 % (45/68) in TBN but only 30 % (34/114) in TOT (p < 0.001) reported that they could read and write.

The majority of respondents (237/378, 63 %) stated to be farmers. Other professions included shopkeeper, forestry, retired, tailor, healer, and others. People who considered themselves as shopkeepers were significantly less likely to participate in the MDA (15/42, 36 %; p = 0.008). 24/42 (57 %) of respondents who stated they were shopkeepers resided in HKT.

### Perceptions of health and malaria specifically

The large majority (295/378; 78 %) of respondents stated unprompted that malaria caused the most health problems in their village followed by respiratory tract infections (56/378; 15 %), diarrhoea (31/378; 8 %) and tuberculosis (6/378; 2 %; Table 2). Eighty nine percent (59/66) of the respondents in TPN and 85 % (104/123) in HKT but only 61 % (69/113) in TOT stated that malaria caused most health problems (p < 0.001).

**Table 2:** Health perceptions in relation to participation

	MDA administration								p value*
	No		Incomplete		Complete		Total		
Number respondents	(56)		(148)		(174)		(378)		
What sickness causes most health problems in your village? (more than one answer was allowed)									
Malaria	42	75 %	116	78 %	137	79 %	295	78 %	0.978
Respiratory tract infections	9	16 %	23	16 %	24	14 %	56	15 %	0.065
Diarrhoea	5	9 %	12	8 %	14	8 %	31	8 %	0.871
Tuberculosis	0	0 %	1	1 %	5	3 %	6	2 %	0.302
What causes malaria? (more than one answer was allowed)									
Mosquitoes transmit malaria	44	79 %	96	65 %	137	79 %	277	73 %	0.012
Don’t know	7	13 %	37	25 %	17	10 %	61	16 %	0.001
No answer	5	9 %	15	10 %	20	11 %	40	11 %	
What do you do to prevent malaria? (more than one answer was allowed)									
Use bed net	38	68 %	107	72 %	137	79 %	282	75 %	0.140
Use mosquito coils	4	7 %	13	9 %	16	9 %	33	9 %	0.854
Cut down the grass	6	11 %	5	3 %	16	9 %	27	7 %	0.249
Spray household with insecticide	4	7 %	8	5 %	8	5 %	20	5 %	0.178
No answer	4	7 %	15	10 %	0		19	5 %	
What kind of complaints do people with malaria have? (more than one answer was allowed)									
Shivering	26	46 %	69	47 %	85	49 %	180	48 %	0.376
Fever	30	54 %	76	51 %	71	41 %	177	47 %	0.268
Headache	25	45 %	64	44 %	86	49 %	175	46 %	0.001
Vomiting	14	25 %	36	24 %	44	25 %	94	25 %	0.046

The table shows the number of respondents who mentioned an answer unprompted. The statistical comparison is between people who mentioned and who didn’t mention an answer. Responses are sorted by totals, percentages % refer to columns not rows

* Chi squared test or Fisher’s exact test

277/378 (73 %) respondents knew that malaria is transmitted by mosquitoes, while 61/378 (16 %) respondents did not know how malaria was transmitted (Table 2). Respondents who didn’t know the causes of malaria were less likely to participate in the MDA than respondents who knew (p = 0.001). Ninety one percent (68/75) of the respondents in KNH, 80 % (98/123) of the respondents in HKT, 83 % (55/66) in TPN were aware of the role of mosquitoes in malaria transmission but only 49 % (56/114) in TOT (p < 0.001).

Three quarters (282/378; 75 %) of the respondents stated unprompted that they used bed nets to prevent malaria. Less than 10 % of the respondents mentioned other methods to prevent malaria such as mosquito coils, cutting grass around the house, or spraying of insecticides (Table 2). Eighty nine percent (67/75) of the respondents in KNH, 76 % (93/123) in HKT but only 62 % (70/113) of the respondents in TOT stated they are using bed nets to prevent malaria (p < 0.001).

The malaria symptoms most frequently mentioned by the respondents were: shivering (180/378, 48 %), fever (177/378, 47 %), headache (175/378, 46 %), and vomiting 94/378, 25 %). There was a statistically significant association between recognizing headache as a potential malaria symptom and participation in the MDA (p = 0.001). There was no statistically significant difference in the proportion of respondents who mentioned fever and shivering as symptoms of malaria between the four villages but more respondents in HKT and TPN than in TOT and KNH knew that malaria can present with headache and vomiting (p = 0.001).

### Understanding the intervention

Eighty five percent (322/378) respondents understood that the purpose of the campaign was to protect against malaria. More than 90 % of the respondents in TPN (62/66) and HKT (113/123) apparently understood this concept but only 70 % (80/114) in TOT (P < 0.001).

The large majority of the respondents indicated that they understood the concept of symptomatic and asymptomatic malaria (Table 3). 224/378 respondents (59 %) correctly agreed that that it can be difficult to identify asymptomatic infected people and 292/378 (77 %) agreed that asymptomatic people can transmit malaria by infecting mosquitoes. This last concept was understood by 88 % (58/66) of the respondents in TPN and 87 % (107/123) of respondents in HKT but only 63 % (71/112) of respondents in TOT (p < 0.001).

**Table 3:** Understanding the intervention in relation to participation

	MDA administration								
	No		Incomplete		Complete		Total		p value*
Number respondents	(56)		(148)		(174)		(378)		
What did you learn and understand during the sensitisation meetings? (more than one answer was allowed)									
Many people who get malaria become sick	49	88 %	125	84 %	159	91 %	333	88 %	0.151
Individuals can have malaria infections and feel perfectly well	16	29 %	63	43 %	81	47 %	324	43 %	0.038
Malaria is more common in the rainy season	42	76 %	114	78 %	146	85 %	305	81 %	0.857
Mosquitoes may become infected from biting individuals who do not get sick	44	80 %	111	75 %	137	79 %	292	78 %	0.672
It is difficult to tell which individuals are carrying malaria without getting sick	36	64 %	92	62 %	96	55 %	224	59 %	0.281
Nothing	1	2 %	1	1 %	1	1 %	3	1 %	0.531
What do you think the medicine is for?									
Protection from malaria	43	77 %	122	82 %	157	90 %	322	85 %	0.023
Gives me strength/energy	1	2 %	8	5 %	8	5 %	17	4 %	0.536
Mosquitoes will not be able to bite me	1	2 %	5	3 %	4	2 %	10	3 %	0.759
After taking the medicine I will not need to sleep under my bed net	0	0 %	1	1 %	0	0 %	1	0 %	0.459
No answer	11	20 %	12	8 %	5	3 %	28	7 %	

The table shows the number of respondents who agree with the answer. The statistical comparison is between people who answered “yes” and people who didn’t. Responses are sorted by totals

* Chi squared test

Reassuringly only a single respondent felt that the participation in the MDA would replace the need for a bed net to protect against mosquito bites.

### Perceptions of the campaign

Most respondents (219/266; 86 %) agreed that it is important that everybody in the village should participate in the intervention irrespective whether the respondent participated or not. Respondents who participated were significantly more likely to state that they had received sufficient information about the campaign (p < 0.001, Table 4). Perhaps not surprisingly participants were also significantly more likely to recommend the program to others than non-participants.

**Table 4:** Perceptions of the campaign in relation to participation

	MDA administration								p value*
	No (n = 22)		Incomplete (n = 96)		Complete (n = 137)		Total (n = 255)		
Do you think it is important for everybody in the village to take the medicine?	16	73 %	78	81 %	125	91 %	219	86 %	0.064
Do you think you received enough information about the MDA?	6	27 %	63	66 %	112	82 %	181	71 %	<0.001
Would you recommend the MDA programme to someone else?	5	23 %	58	60 %	103	75 %	166	65 %	<0.001

The table shows the number of respondents who agree with the answer. The statistical comparison is between people who answered “yes” and people who didn’t. Responses are sorted by totals

* Chi squared test or Fisher’s exact test

### Independent factors associated with participation in the MDA

A multivariate logistic regression model identified two factors which were independently associated with participation in the MDA (Table 5). Respondents living in KNH and TPN were significantly more likely to participate than respondents from HKT or TOT. Secondly respondents who felt they had received sufficient information about the campaign were significantly more likely to participate than respondents who didn’t have this impression.

**Table 5:** Multivariate analysis of key variables associated with participation in the campaign

	MDA								
	Less than complete		Complete		Total	OR (univariate)		OR (multivariate)*	
	n	%	n	%					
Number respondents	204	54	174	46	378		p value		p value
Demographics of respondents									
Village									
HKT	86	70	37	30	123	1		1	
KNH	17	23	58	77	75	7.9	<0.001	17.3*	0.001*
TOT	78	68	36	32	114	1.1	0.803	2.7*	0.2*
TPN	23	35	43	65	66	4.4	<0.001	9.3*	0.007*
Ethnicity									
Burman	59	67	29	33	88	1		1	
Other	3	37	5	63	8	3.4	0.110	1.5*	0.6*
Paw	42	39	66	61	108	3.2	<0.001	2.0*	0.03
Sgaw	99	58	73	42	172	1.5	0.139	1.9*	0.08*
Profession: shop-keeper									
No	175	52	161	48	336	1		1	
Yes	29	69	13	31	42	0.5	0.041	0.6**	0.1**
Health perceptions in relation to participation: do you know what causes malaria?									
I know	160	50	157	50	317	1			
I don’t know	44	72	17	28	61	0.4	0.002	0.5*	0.05*
Perceptions of the campaign: do you think you received enough information about the MDA?									
Yes	69	38	112	62	181	1		1	
No	20	53	18	47	38	0.6	0.1	0.5**	0.07**
Don’t know	29	81	7	19	36	0.2	<0.001	0.3**	0.01**
Would you recommend the MDA programme to someone else?									
Yes	63	38	103	62	166	1		1	
No	2	100	0	0	2	NA		NA	
Don’t know	53	61	34	39	87	0.4	0.001	0.9**	0.9**

The most relevant key variables in each table which were statistically significant in the univariate analysis were included in the multivariate analysis

* Adjusted for village, ethnicity, do you know what causes malaria, and how did you hear about malaria—study staff

** Adjusted for village only

### Comparing reasons for non-participation

**Fig. 5:**
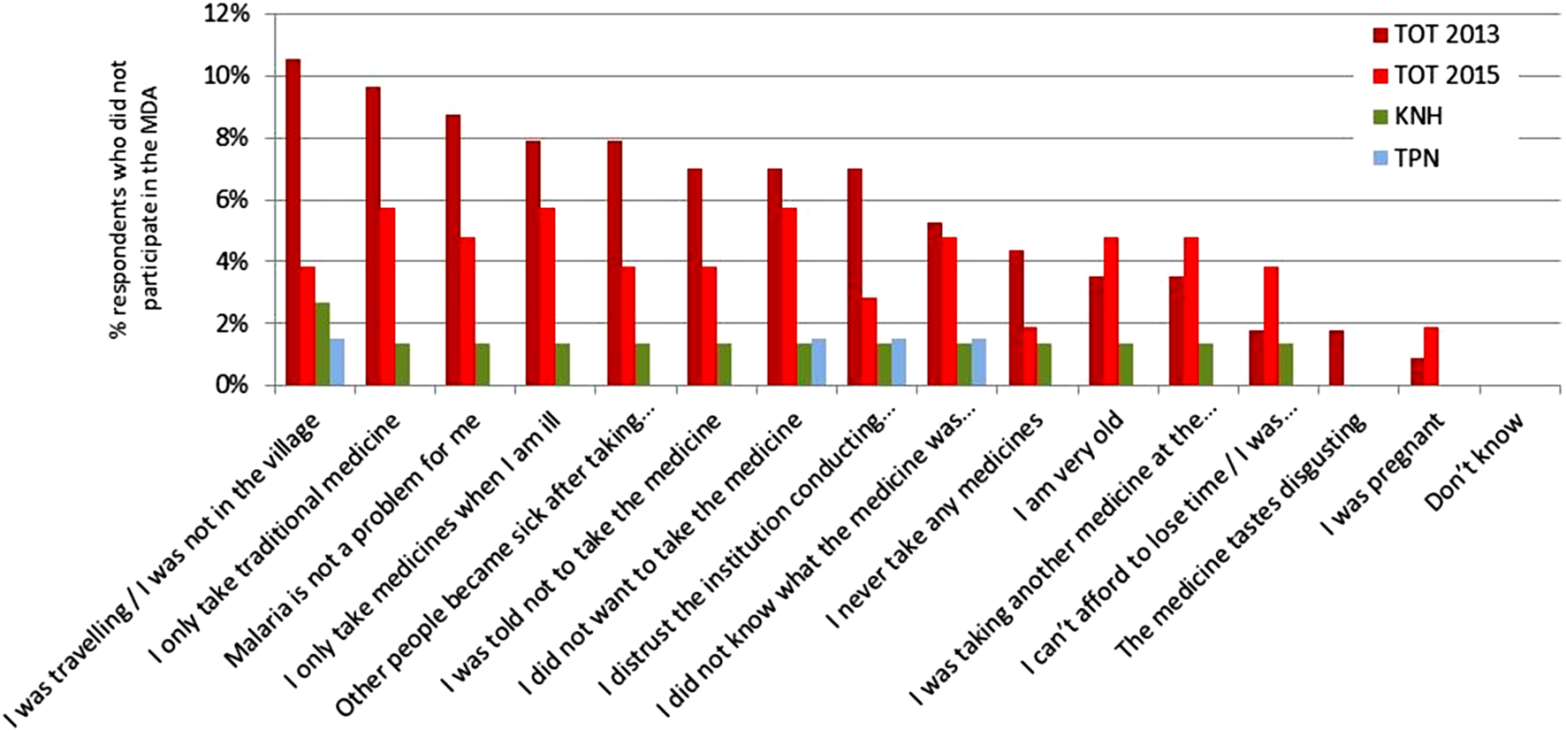
Reasons for non participation given by the respondents who didn’t participate (by village, no data available for HKT)

The reasons for non-participation are compared between three villages TOT, KNH, and TPN as well as following a second MDA in in TOT in 2015 (no data for HKT available; Fig. 5). In all villages the most frequently mentioned reason for non-participation was absence from the village at the time of the campaign. 12/19 (63 %) respondents who didn’t participate in TOT in 2013 used this explanation compared to only 4/21 (19 %) respondents in 2015 (p < 0.001). Also distrust which was mentioned by 8/19 (42 %) of participants in 2013 had dropped to 3/21 (14 %) by 2015 (p = 0.05).

### Changes in malaria knowledge in TOT between 2014 and 2015

The respondents’ understanding of the intervention and knowledge of malaria had overall improved between the first set of interviews was conducted in TOT in February 2014 and the second set conducted 16 months later in June 2015 (Table 6). The number of respondents who didn’t know what causes malaria had significantly dropped from 38 % (43/114) in 2014 to 23 % (25/109) in 2015 (p = 0.017). More respondents were aware that malaria symptoms include headache 36 % (40/112) in 2014 to 51 % (54/107) in 2015 (p = 0.016) and fever 44 % (50/113) in 2014 to 55 % (59/107) in 2015 (p = 0.041). Also the percentage of respondents who understood the importance of all villagers participating in the MDA had significantly increased from 70 % (78/112) in 2014 to 88 % (94/107) in 2015 (p = 0.001). Only the appreciation of asymptomatic malaria had significantly decreased 48 % (54/112) in 2014 to 29 % (31/107) in 2015 (p = 0.003).

**Table 6:** Significant changes in understanding the intervention and knowledge of malaria in one study village (TOT) over 1 year

	2014	2015	p
What causes malaria?			
Mosquitoes transmit malaria	56/114 (49 %)	60/109 (55 %)	0.376
Don’t know	43/114 (38 %)	25/109 (23 %)	0.017
What sickness causes most health problems in your village?			
Malaria	69/113 (61 %)	80/107 (75 %)	0.078
Respiratory tract infections	15/113 (13 %)	28/107 (26 %)	0.009
What kind of complaints do people with malaria have?			
Fever	50/113 (44 %)	59/107 (55 %)	0.041
Headache	40/112 (36 %)	54/107 (51 %)	0.016
What did you learn and understand during the sensitisation meetings?			
Many people who get malaria become sick	84/112 (75 %)	102/107 (95 %)	<0.001
Individuals can have malaria infections and feel perfectly well	54/112 (48 %)	31/107 (29 %)	0.003
Mosquitoes may become infected from biting individuals who do not get sick	71/112 (63 %)	81/107 (76 %)	0.048
Do you think it is important for everybody in the village to take the medicine?			
Agree	78/112 (70 %)	94/107 (88 %)	0.001

## Discussion

The study found that overall 83 % of respondents took at least three consecutive doses of anti-malarials which are essential to completely clear *P. falciparum* infections. The participation was highly heterogeneous from 71 % (in HKT) to as high as 96 % (in KNH). A range of factors was found to be associated with participation in the MDA. The respondents from two villages KNH and TPN were much more likely to participate in the MDA than respondents from the other two villages HKT and TOT. While the more compliant villages KNH and TPN gave the appearance of cohesive communities the villages with low participation TOT and HKT were unique in their own ways.

In TOT the community is historically divided between two armed factions. The village has grown together from two separate villages but the population remains divided. Uniting the population in participating in the intervention has not been successful. In the absence of a cohesive community an intervention supported by one faction tends to be opposed by the other faction irrespective of potential benefits. The respondents from TOT gave different answers than the respondents from the other three villages suggesting different believes and perceptions. Their knowledge regarding the cause, signs and symptoms of malaria as well as their understanding of the intervention was significantly lower than in the other villages. The poor understanding of the disease as well as the rationale for the intervention to eliminate malaria has probably contributed to the low participation of respondents. The MDA in 2014 had been well tolerated and no severe adverse events attributable to the study drugs had been reported which should have diffused any safety concerns. But there were unconfirmed rumours about adverse events unfairly blamed on the anti-malarial drugs in some parts of the village. The investigators attributed the continued transmission of falciparum malaria after the MDA to the relatively poor participation resulting in a residual parasite reservoir. Hence the MDA was repeated at the end of the 2-year surveillance period in 2015. The study staff had a continued presence over the 2-year surveillance period. The increased knowledge of malaria in 2015 compared to 2014 may reflect the impact of the education efforts provided by the study team. Yet increased knowledge and understanding did not translate in higher participation rates during the second MDA in 2015 suggesting that in TOT the historical division and antagonism between fractions of the population played a more important role in non-participation than lack of understanding.

The other village with low participation rates was HKT. HKT shares with TOT a fragmented community. The population of HKT has rapidly increased over the last decade and many of the recent arrivals consider themselves temporary visitors and hold different believes than the indigenous residents. Because they plan to stay only for a limited period the newcomers don’t consider themselves necessarily as part of the community and see little reason to participate in a campaign, which does not provide direct benefits. In HKT the relatively high refusal rate in the MDA was associated with Burman ethnicity. Specifically Burman shopkeepers felt little need to participate in the MDA. Participating in the campaign requires closing the shop for nine mornings to come to the healthcare centre. The absence of a direct benefit is hence potentially compounded by a loss of income. The members of the Burman minority in these tribal areas see themselves at low risk for malaria, are generally more affluent and should they become sick with malaria will have easier access to appropriate healthcare than the indigenous villagers.

An alternative, more universal reason for non-participation in the MDA independent of village was related to an inadequate understanding of the intervention. Respondents who felt that they had received sufficient information were significantly more likely to participate than responds who felt that they didn’t know enough about the campaign. The perception of being well informed about the intervention has been shown to play a critical role in other mass drug administrations against malaria [10] and other infectious diseases including lymphatic filariasis [14]. But there was no direct correlation between understanding and willingness to participate in the campaign. Many community members had limited understanding of the intervention or knowledge of malaria but still participated. On the other hand relatively well informed and educated community members such as the shopkeepers in HKT refused to participate.

The study relied on recollection and opinions, which may be biased and inaccurate. The most frequent response to the question why they did not participate in the MDA was “I was travelling/I was not in the village at the time.” This reply is more polite than stating “I distrust the institution conducting the campaign” even though distrust could have played a role in the decision to be absent from the campaign. To get a more detailed understanding of the true reasons for incomplete or non-participation including deeper motivations, fears and apprehension, in-depth interviews and focus group discussions will be needed.

The biologic principles underlying the drug administrations are complex and require a relatively sophisticated understanding of the pathogenesis of malaria, the concept of a parasite reservoir and the treatment of subclinical infections. These concepts are not easy to communicate especially over a short time period in a field setting to semi-literate communities few of whom have a secondary education. In the absence of a comprehensive understanding of the risks and benefits of the campaigns many villagers have to decide whether or not to participate in the campaign based on their trust in the study staff who have explained the intervention, the potential risks and benefits. Several researchers have explored the concept of trust in decisions whether or not to participate in public health interventions including clinical trials [15–18]. Qualitative research has shown time and again that trust plays a central role in the uptake of public health interventions. Trust relationships tend to be complex. In some settings it is not the promised indirect benefit of intervention such as the elimination of a disease but the tangible direct benefits such as free appropriate primary health care and free transport to a hospital in an emergency at least for the duration of the surveillance period [15]. In the absence of tangible benefits and limited comprehension of the broader indirect benefits for the community it is essential for the potential participant to be able to believe that the intervention team will act in the best interest of the participant. To gain such trust requires time and persistent good will from both sides. The repeated campaigns in TOT illustrate in a fragmented community 2 years of permanent presence, primary health care provision and successful health education were not enough to increase participation.

## Conclusions

The study found excellent participation in two cohesive communities. In contrast in fragmented communities attempts to mobilise the entire community were less successful. The findings suggest several approaches how community participation in anti-malarial mass drug administrations can be increased. Understanding the purpose and the reasons underlying the intervention is a helpful but perhaps not essential pre-condition for participation. Time invested in information campaigns is a productive investment to increase coverage. Not only is the information provided critical but how this information is communicated is essential. A sincere effort to provide honest information to community members can be one of several steps to build trust with the community. Building trust in a community is a complex, time consuming undertaking but only if all community members are convinced that they will ultimately benefit from the campaign can the very high participation rates achieved required for the elimination of infectious diseases like malaria. Based on the experience in one fragmented village overcoming internal divisions within a village is an extraordinary challenge requiring additional time, engagement and repeated campaigns.
